# An interpretable MRI radiomics approach for preoperative prediction of lymphovascular space invasion in cervical cancer using optimal peritumoral region

**DOI:** 10.3389/fonc.2026.1859587

**Published:** 2026-08-31

**Authors:** Xianyan Wu, Chuanfang Xu, Shan Shi, Chengbin Ye, Qun Zhong, Wenjie Yan

**Affiliations:** Department of Radiology, The Affiliated People’s Hospital of Fujian University of Traditional Chinese Medicine, Fuzhou, China

**Keywords:** cervical cancer, lymphovascular space invasion, machine learning, peritumoral region, radiomics, Shapley additive explanations

## Abstract

**Background:**

Lymphovascular space invasion (LVSI) is an important pathological feature associated with tumor aggressiveness and adverse prognosis in cervical cancer. However, reliable preoperative prediction of LVSI remains a challenge. This study aimed to develop and validate an MRI-based radiomics model incorporating intratumoral and peritumoral features for LVSI prediction and systematically evaluate the optimal peritumoral extent.

**Materials and methods:**

In this single-center study comprising both retrospective and prospective cohorts, 204 patients with pathologically confirmed cervical cancer were randomly divided into a training cohort (n = 142) and a test cohort (n = 62). Radiomics features were extracted from the intratumoral and peritumoral regions with expansion distances of 1, 3, and 5 mm. Feature selection was performed using intraclass correlation coefficient (ICC) analysis, minimum redundancy maximum relevance (mRMR), and least absolute shrinkage and selection operator (LASSO) analysis. Six machine learning algorithms, including logistic regression [LR], support vector machine, random forest, ExtraTrees, LightGBM, and multilayer perceptron, were used to construct the predictive models. The model performance was evaluated using receiver operating characteristic analysis, calibration curves, and decision curve analysis. SHapley Additive Explanations (SHAP) were applied to interpret the optimal models.

**Results:**

Among all ROI configurations, the Intra+P1 model demonstrated the best overall performance, particularly when it was combined with LR. The LR-based Intra+P1 model achieved an AUC of 0.866 (95% CI: 0.807–0.926) in the training cohort and 0.843 (95% CI: 0.731–0.955) in the test cohort respectively. Increasing the peritumoral expansion from 1 mm to 3 mm or 5 mm did not further improve predictive performance. The addition of clinical variables (tumor diameter and SCC level) did not provide a significant incremental predictive value. Calibration and decision curve analyses demonstrated good agreement and favorable clinical utility of the model. SHAP analysis showed that both intratumoral and peritumoral features contributed substantially to the model prediction, with texture features playing a dominant role.

**Conclusion:**

This interpretable MRI-based radiomics model facilitates accurate preoperative prediction of LVSI in cervical cancer. A narrowly defined 1-mm peritumoral region provides the most informative complementary information, underscoring the importance of the tumor-invading front. This approach offers a noninvasive tool for risk stratification and may support individualized treatment decision-making.

## 1 Introduction

Cervical cancer remains a major global health burden, particularly in low- and middle-income regions, where both incidence and mortality continue to be substantial despite advances in screening and treatment strategies (1–3). In clinical practice, treatment selection depends heavily on the accurate assessment of tumor aggressiveness; however, achieving reliable preoperative risk stratification remains challenging.

Lymphovascular space invasion (LVSI) is widely recognized as an important marker of tumor invasiveness in cervical cancer and is closely associated with unfavorable clinical outcomes (4). Its presence is associated with a higher likelihood of lymph node metastasis, increased recurrence risk, and poorer survival, highlighting its clinical significance (5, 6). Accordingly, LVSI has been incorporated into risk stratification frameworks and is routinely considered in prognostic assessments and treatment planning (7).

Currently, LVSI can only be confirmed through postoperative histopathological examination, which limits its availability during preoperative decision-making. Attempts to estimate LVSI status using conventional imaging findings or clinical parameters have yielded suboptimal results (8, 9), suggesting that existing approaches are inadequate. Therefore, the development of a reliable and noninvasive method for preoperative prediction is essential to enable more precise risk stratification and individualized treatment planning.

Radiomics converts medical images into quantitative features that reflect tumor phenotypes and heterogeneity beyond visual assessment (10–12). By capturing subtle imaging patterns, radiomics-based models have shown potential in evaluating tumor aggressiveness, predicting treatment response, and estimating clinical outcomes across multiple cancer types, including cervical cancer (13).

Previous radiomics studies have largely focused on intratumoral regions, where features are extracted from the tumor itself. However, tumor behavior is shaped not only by intrinsic properties but also by interactions at the tumor–host interface. The peritumoral area, representing this interface, may capture imaging correlates of invasion, angiogenesis, inflammatory activity, and stromal remodeling (14–16). Therefore, incorporating information from this region into radiomics models may provide additional value for improving predictive performance.

An important unresolved issue in peritumoral radiomics is the definition of the optimal spatial extent for the extraction of features. If the peritumoral region is defined too narrowly, relevant microenvironmental information may be missed. Conversely, excessive expansion may introduce signals from adjacent normal tissues, thereby compromising the model’s specificity and robustness. Consequently, the relationship between peritumoral extent and predictive performance remains insufficiently characterized and represents a key methodological challenge.

In this study, we developed and validated an MRI-based radiomics model for the preoperative prediction of LVSI in cervical cancer. By integrating features derived from both intratumoral and peritumoral regions across multiple expansion distances (1, 3, and 5 mm), we further examined how the peritumoral scale influences model performance and aimed to determine the most informative spatial range for LVSI prediction. Unlike previous studies that adopted a single empirically defined peritumoral region, we systematically compared multiple peritumoral extents within a unified multiparametric MRI radiomics framework to identify the optimal spatial scale for LVSI prediction.

## 2 Materials and methods

### 2.1 Study population

The study protocol complied with the Declaration of Helsinki and was approved by the Institutional Review Board of the Affiliated People’s Hospital of Fujian University of Traditional Chinese Medicine (approval numbers: 2025-051–01 for the retrospective cohort and 2023-019–01 for the prospective cohort). The requirement for informed consent was waived for the retrospective cohort, while written informed consent was obtained from participants in the prospective cohort.

This single-center study comprised both retrospective and prospective cohorts. Patients with pathologically confirmed cervical cancer who underwent surgical treatment between January 2015 and February 2026 were consecutively included in the study. The inclusion criteria were as follows: (1) histopathological confirmation of cervical cancer; (2) preoperative pelvic MRI performed within 2 weeks before surgery; (3) sufficient image quality and complete clinicopathological data; (4) absence of other malignancies; and (5) no prior antitumor treatment (including chemotherapy, radiotherapy, or cervical conization) before MRI examination. The exclusion criteria were as follows: (1) incomplete clinical or pathological data; (2) presence of other malignancies; (3) receipt of any preoperative treatment; and (4) poor image quality or severe artifacts.

A total of 204 patients were finally included and randomly divided into a training cohort (n = 142) and a test cohort (n = 62) using a stratified sampling approach at a ratio of 7:3.

The clinical variables collected included demographic and clinical characteristics [age, clinical symptoms (contact vaginal bleeding and abnormal discharge), and human papillomavirus (HPV) status], as well as serum tumor markers, including squamous cell carcinoma antigen (SCC), carcinoembryonic antigen (CEA), carbohydrate antigen 125 (CA125), and carbohydrate antigen 19-9 (CA19-9). These tumor markers were dichotomized into elevated and normal levels based on the clinical reference values. The maximum tumor diameter was measured using preoperative MRI. LVSI status was determined based on postoperative histopathological findings and used as the reference standard.

### 2.2 MRI acquisition

All patients underwent pelvic MRI examinations using 3.0-T scanners (MAGNETOM Verio, Siemens Healthineers; Ingenia CX, Philips Healthcare) equipped with phased-array body coils.

The MRI protocol included axial T1-weighted imaging (T1WI), axial T2-weighted imaging (T2WI), fat-suppressed T2-weighted imaging (FS-T2WI), diffusion-weighted imaging (DWI), and sagittal T2WI sequences. Dynamic contrast-enhanced MRI was performed after intravenous administration of gadolinium-diethylenetriamine pentaacetic acid (Gd-DTPA) at a dose of 0.1 mmol/kg and an injection rate of 2.5 mL/s.

The detailed imaging parameters, including the repetition time (TR), echo time (TE), field of view (FOV), slice thickness, and interslice gap, are provided in **Supplementary Table 1**.

### 2.3 Tumor segmentation, peritumoral region construction, and feature extraction

Tumor segmentation was manually performed on T2-weighted imaging (T2WI), apparent diffusion coefficient (ADC) maps, and contrast-enhanced T1-weighted imaging (CE-T1WI) independently by experienced radiologists using 3D Slicer software (version 5.6.2). For each sequence, the entire tumor volume was manually delineated slice-by-slice to generate an intratumoral ROI. The intratumoral ROI was delineated directly on the corresponding images in a sequence-specific manner, and subsequent peritumoral expansion was performed based on the sequence-specific intratumoral ROI after preprocessing.

Prior to peritumoral ROI construction and radiomics feature extraction, all MRI images were subjected to standardized preprocessing to improve feature reproducibility. Specifically, N4 bias field correction was applied to reduce the low-frequency intensity nonuniformity caused by MRI field inhomogeneity. Subsequently, T2WI, ADC maps, and CE-T1WI were resampled to an isotropic voxel size of 1 × 1 × 1 mm³ to ensure spatial consistency across patients and MRI sequences. Linear interpolation was used for MRI images, whereas nearest-neighbor interpolation was applied to the segmentation masks to preserve label integrity. Gray-level normalization was then performed to reduce inter-patient and inter-sequence intensity variability before the radiomics feature extraction.

Based on the intratumoral ROI, peritumoral regions were generated by isotropic outward expansion of 1, 3, and 5 voxels, corresponding to physical distances of 1, 3, and 5 mm, respectively, after image resampling to isotropic 1 × 1 × 1 mm³ voxel spacing. Because all images were spatially standardized before the ROI expansion, the voxel-based expansion was equivalent to the millimeter-based expansion in the present study. This resulted in four ROI configurations: intratumoral (Intra), intratumoral plus 1-mm peritumoral region (Intra+P1), intratumoral plus 3-mm peritumoral region (Intra+P3), and intratumoral plus 5-mm peritumoral region (Intra+P5).

After the automatic generation of the expanded peritumoral ROIs, all masks were visually reviewed by radiologists. Obvious non-target pelvic structures, particularly the bladder lumen and rectal lumens, were manually excluded from the expanded regions. However, anatomically contiguous adjacent pericervical or uterine stromal tissues were retained, as these regions may represent a biologically relevant tumor–host interface associated with LVSI. Radiomics features were extracted from T2WI, ADC, and CE-T1WI sequences, including first-order statistics, shape features, and texture features (gray-level co-occurrence matrix, gray-level run-length matrix, and gray-level size zone matrix). A total of 1105 features were extracted from each MRI sequence, resulting in 3315 features for each ROI configuration across the three MRI sequences. Feature extraction was performed using PyRadiomics (version 3.0.1) implemented in Python (version 3.7.12).

### 2.4 Feature selection and model construction

To ensure feature reproducibility and robustness, intra- and interobserver agreement was assessed using the intraclass correlation coefficient (ICC). Only features with ICC ≥ 0.75 were retained for subsequent analysis.

To reduce feature redundancy, Pearson’s correlation analysis was performed, and features with an absolute correlation coefficient > 0.9 were removed. Subsequently, the minimum redundancy maximum relevance (mRMR) method was applied to rank features, and the top 20 features were selected. Further dimensionality reduction was performed using least absolute shrinkage and selection operator (LASSO) regression with cross-validation to determine the optimal regularization parameter.

**Figure 1:**
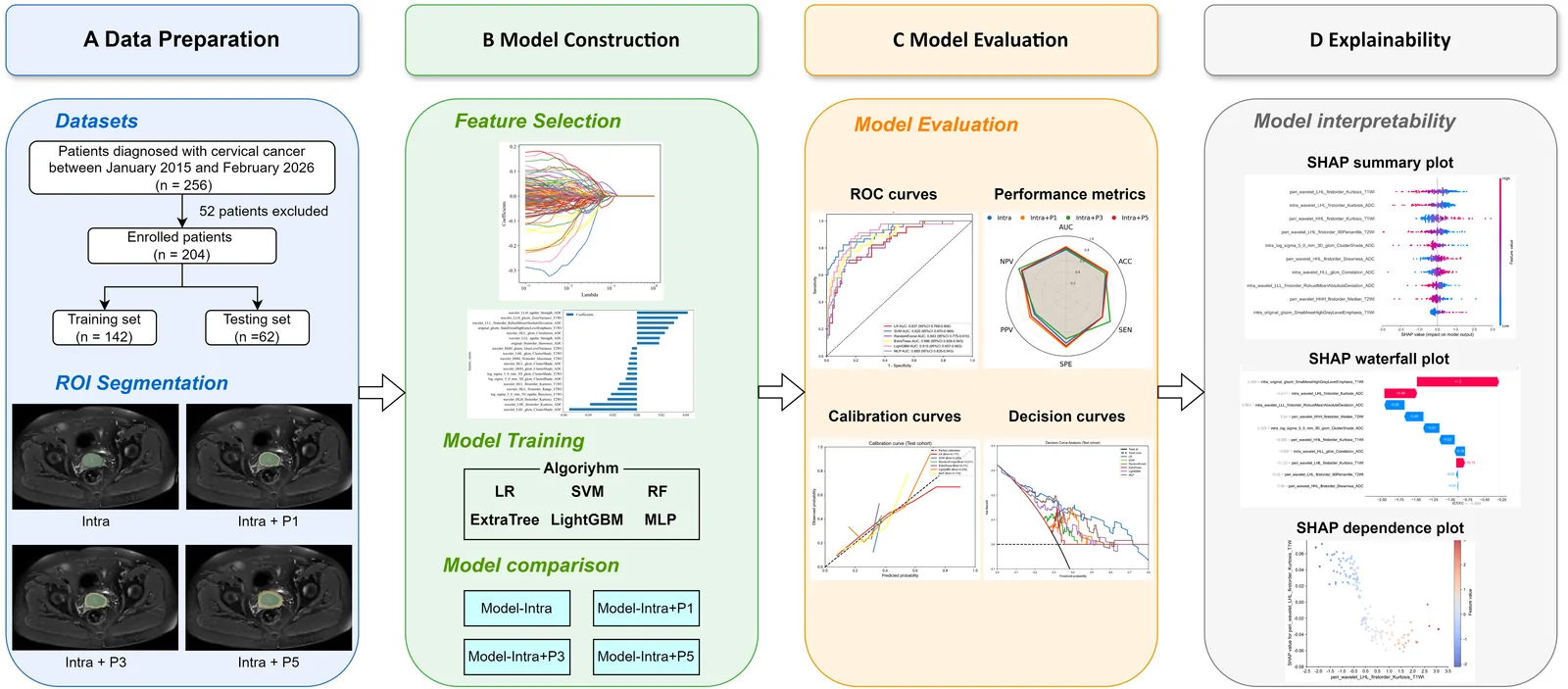
Workflow of the radiomics analysis for predicting lymphovascular space invasion (LVSI) in cervical cancer.

Radiomics signatures were constructed using the selected features. Multiple machine learning classifiers—including logistic regression (LR), support vector machine (SVM), random forest (RF), Extra Trees (ET), light gradient boosting machine (LightGBM), and multilayer perceptron (MLP)—were subsequently applied to develop predictive models, with the aim of identifying the best-performing approach. The overall radiomics workflow, including tumor segmentation, peritumoral ROI construction, feature extraction, feature selection, model development, performance evaluation, and model interpretation, is illustrated in **Figure 1**.

### 2.5 Model evaluation

Model performance was primarily quantified using receiver operating characteristic (ROC) analysis, with the area under the curve (AUC) and corresponding 95% confidence intervals reported. Additional indicators, including accuracy, sensitivity, specificity, positive predictive value (PPV), negative predictive value (NPV), and F1-score, were calculated to provide a comprehensive assessment. Calibration was examined using calibration curves, and clinical utility was assessed using decision curve analysis (DCA).

To identify the clinical predictors associated with LVSI, univariate logistic regression analysis was first performed, followed by multivariate analysis incorporating variables with statistical significance. The factors that remained significant in the multivariate model were considered independent predictors.

A combined model was subsequently established by integrating radiomics features from the optimal ROI configuration and independent clinical variables. The performance of the intratumoral, optimal radiomics, and combined models was compared. In addition, different ROI configurations and classifiers were evaluated simultaneously.

Finally, the distribution of predicted probabilities in the test cohort was visualized using box-and-whisker plots to assess the risk stratification capability, with the optimal cutoff value determined based on the Youden index.

### 2.6 Model interpretability

Model interpretability was explored using Shapley Additive Explanations (SHAP) applied to the best-performing model. SHAP values were computed to quantify the contribution of each feature to the model predictions, allowing interpretation at both the population and individual levels.

At the population level, feature importance was ranked according to the mean absolute SHAP values and visualized using bar plots, while summary (beeswarm) plots illustrated the distribution and directional effects of features across the dataset.

At the individual level, waterfall and force plots were used to explain model predictions for representative cases by demonstrating the positive and negative contributions of features. Dependence plots were used to examine the relationships between key features and their corresponding SHAP values. Decision plots were generated to visualize the cumulative contribution of features across samples and provide insights into model decision pathways.

### 2.7 Statistical analysis

Statistical analyses were conducted using Python (version 3.7.12) and R (version 4.2.2). Continuous variables are presented as mean ± standard deviation or median (interquartile range), depending on the data distribution, and were compared using the independent-samples t-test, or Mann–Whitney U test as appropriate. Categorical variables are reported as counts (percentages) and were analyzed using the chi-square or Fisher’s exact test.

To identify factors associated with LVSI, univariate logistic regression analysis was initially performed, followed by multivariate analysis, including variables that reached statistical significance. The results are reported as odds ratios (ORs) with 95% confidence intervals (CIs).

Model discrimination was assessed based on receiver operating characteristic (ROC) analysis, with the area under the curve (AUC) and corresponding 95% confidence intervals calculated using the DeLong method. Pairwise comparisons of ROC AUCs between competing ROI configurations and machine learning models were performed using the DeLong test based on an independent testing cohort. The calibration performance was examined using calibration curves and the Hosmer–Lemeshow goodness-of-fit test, together with the Brier score to quantify predictive accuracy.

The clinical utility was further evaluated using decision curve analysis (DCA) across a range of threshold probabilities. All statistical tests were two-sided, and a P-value < 0.05 was considered statistically significant.

Bootstrap sensitivity analysis was performed to assess the robustness of the final radiomics model. Specifically, 1,000 bootstrap resampling iterations were conducted in the testing cohort, and the AUC was calculated for each iteration. The mean bootstrap AUC and corresponding 95% confidence intervals were subsequently estimated to evaluate the stability of the model performance.

## 3 Results

### 3.1 Clinical and pathological characteristics

A total of 204 patients with pathologically confirmed cervical cancer were included, comprising 82 patients with LVSI (40.2%) and 122 without LVSI (59.8%).

The training and test cohorts comprised 142 and 62 patients, respectively. The distribution of clinical characteristics between the two cohorts is presented in **Table 1**.

**Table 1:** Baseline clinical characteristics of patients in the training and testing cohorts.

Characteristic	Training cohort (n=142)	Testing cohort (n=62)	t/χ²	P-values
Age (years)	55.8 ± 10.93	55.98 ± 11.45	0.111	0.911
Maximum tumor diameter (cm)	3.27 ± 1.28	3.46 ± 1.48	0.911	0.364
Clinical symptoms			0.066	0.797
Absent	10 (7.0%)	5 (8.1%)		
Present	132 (93.0%)	57 (91.9%)		
HPV status			5.403	0.020*
Negative	81 (57.0%)	46 (74.2%)		
Positive	61 (43.0%)	16 (25.8%)		
SCC (ng/ml)			0.492	0.483
<2.0	68 (47.9%)	33 (53.2%)		
≥2.0	74 (52.1%)	29 (46.8%)		
CEA (μg/L)			1.728	0.189
<5.0	110 (77.5%)	53 (85.5%)		
≥5.0	32 (22.5%)	9(14.5%)		
CA19-9 (U/ml)				0.235
<30.0	130 (91.5%)	60 (96.8%)		
≥30.0	12 (8.5%)	2 (3.2%)		
CA125 (U/ml)			1.634	0.201
<35.0	128 (90.1%)	52 (83.9%)		
≥35.0	14 (9.9%)	10 (16.1%)		

Data are presented as mean ± standard deviation or number (percentage). Continuous variables were compared using Student’s t test. Categorical variables were compared using the chi-square test or Fisher’s exact test, as appropriate. Fisher’s exact test was used when expected frequencies were less than 5, and only P-values are reported. LVSI-positive/negative cases were 57/85 in the training cohort and 25/37 in the testing cohort.

Univariate logistic regression analysis demonstrated that maximum tumor diameter and serum squamous cell carcinoma antigen (SCC) levels were significantly associated with LVSI status (both P < 0.05). These variables remained independent predictors in the multivariate analysis (both P < 0.05), as detailed in **Table 2**.

**Table 2:** Univariate and multivariate logistic regression analyses of clinical variables for predicting lymphovascular space invasion (LVSI) in patients with cervical cancer.

Characteristic	Univariate logistic regression		Multivariate logistic regression	
	OR (95% CI)	P-values	OR (95% CI)	P-values
Age	1.014 (0.988-1.040)	0.284		
Maximum tumor diameter	1.374 (1.102-1.713)	0.005*	1.270 (1.010-1.598)	0.041*
Clinical symptoms (present vs absent)	4.364 (0.950-20.039)	0.058		
HPV status (positive vs negative)	1.546 (0.869-2.749)	0.138		
SCC (elevated vs normal)	2.873 (1.605-5.145)	<0.001*	2.474 (1.357-4.514)	0.003*
CEA (elevated vs normal)	1.371 (0.688-2.733)	0.370		
CA19-9 (elevated vs normal)	1.125 (0.375-3.371)	0.833		
CA125 (elevated vs normal)	1.901 (0.807-4.481)	0.142		

OR, odds ratio; CI, confidence interval; HPV, human papillomavirus; SCC, squamous cell carcinoma antigen; CEA, carcinoembryonic antigen; CA125, cancer antigen 125; CA19-9, carbohydrate antigen 19-9. *P < 0.05 was considered statistically significant.

### 3.2 Feature selection and radiomics signature construction

A total of 3,315 radiomics features were initially extracted from each ROI configuration (Intra, Intra+P1, Intra+P3, and Intra+P5). Among these, 3,039, 2,938, 3,037, and 3,039 features demonstrated good reproducibility (intra- and interobserver ICC ≥ 0.75).

After feature selection, the Intra+P1 model retained 8 intratumoral and 12 peritumoral features. Similarly, the Intra+P3 model retained 8 intratumoral and 12 peritumoral features, whereas the Intra+P5 model retained 8 intratumoral and 12 peritumoral features. Radiomics signatures were constructed based on the selected features. The top 20 features for each model are summarized in **Supplementary Table 2**.

### 3.3 Performance of machine learning models

**Figure 2:**
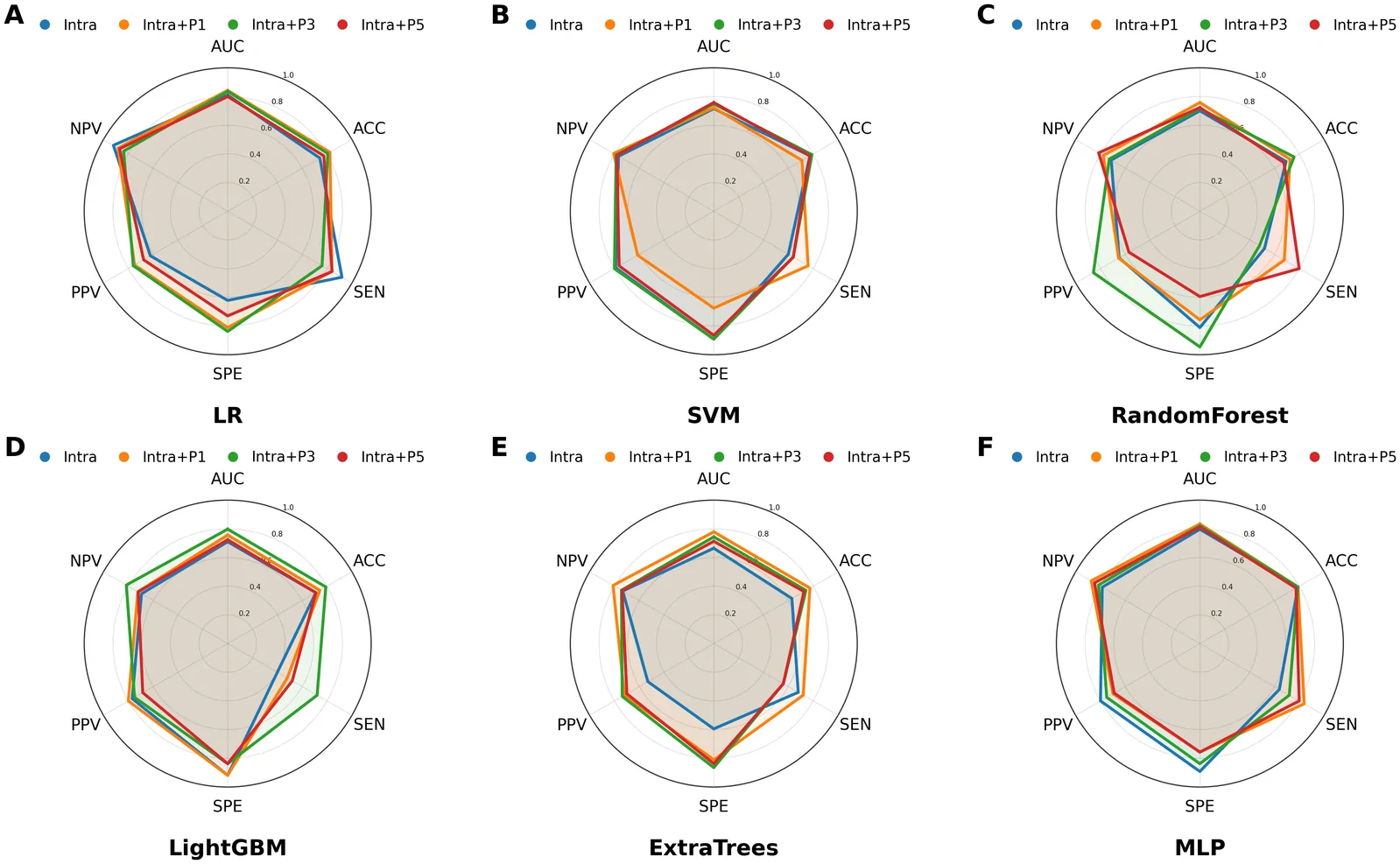
Radar plots illustrating the predictive performance of six machine learning classifiers across different tumor regions (intratumoral and peritumoral; Intra, Intra+P1, Intra+P3, and Intra+P5) in the test cohort. **(A)** Logistic regression (LR); **(B)** support vector machine (SVM); **(C)** random forest (RF); **(D)** LightGBM; **(E)** extra trees (ET); and **(F)** multilayer perceptron (MLP). Each radar plot summarizes multiple performance metrics, including area under the receiver operating characteristic curve (AUC), accuracy (ACC), sensitivity (SEN), specificity (SPE), positive predictive value (PPV), and negative predictive value (NPV).

Radiomics-based predictive models for LVSI were developed using six machine learning classifiers (LR, SVM, RF, ET, LightGBM, and MLP) across four ROI configurations (Intra, Intra+P1, Intra+P3, and Intra+P5). A radar plot (**Figure 2**) presents the distribution of the performance metrics and highlights the overall balance among the models across different configurations.

The Intra+P1 configuration demonstrated the best overall performance across all models, with LR achieving the highest results. The AUC was 0.866 (95% CI: 0.807–0.926) in the training cohort and 0.843 (95% CI: 0.731–0.955) in the test cohort.

**Figure 3:**
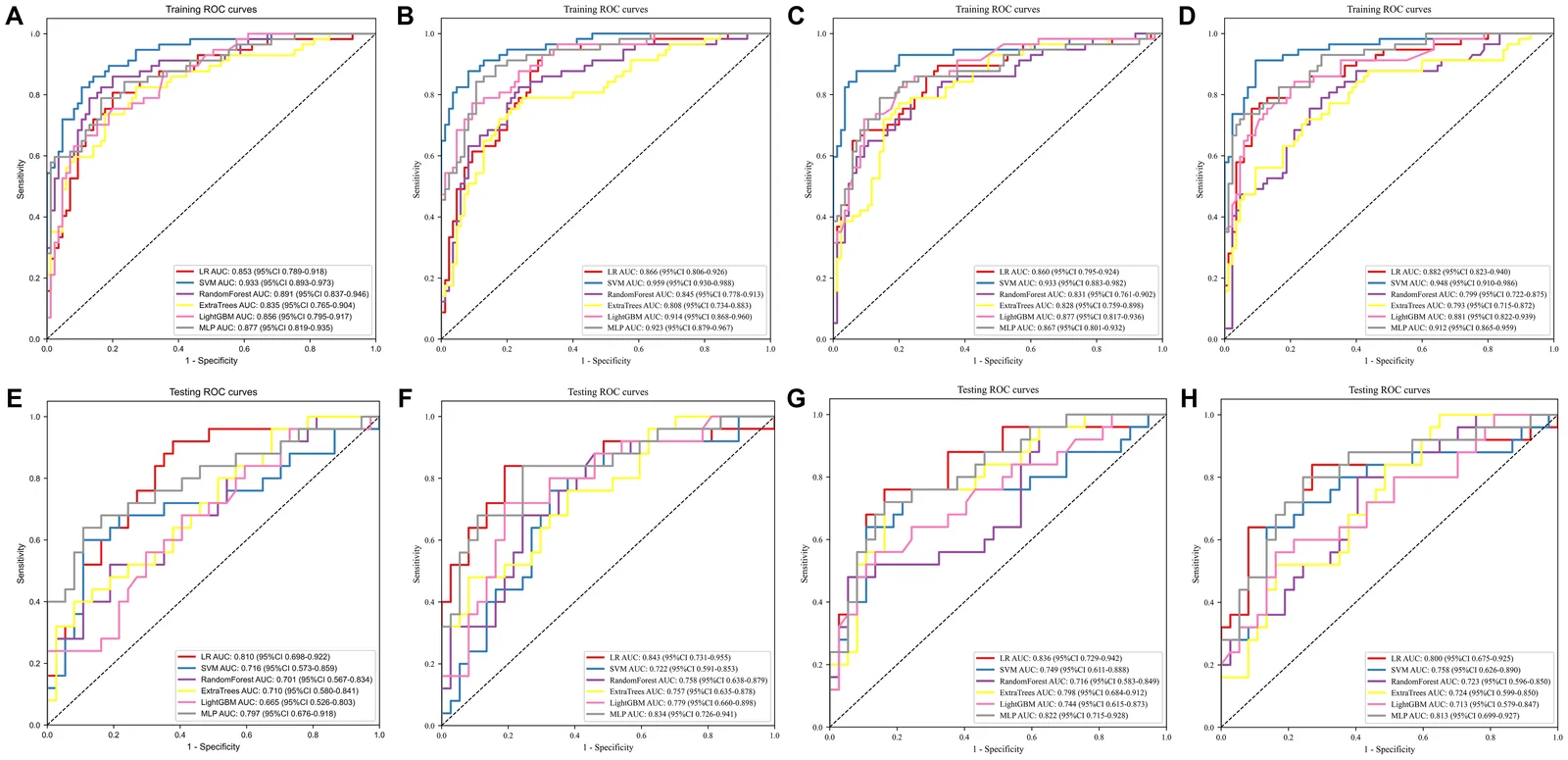
Receiver operating characteristic (ROC) curves of machine learning models across different tumor regions (intratumoral and peritumoral) in the training and test cohorts. **(A)** Intratumoral (Intra) model in the training cohort; **(B)** Intra+P1 model in the training cohort; **(C)** Intra+P3 model in the training cohort; **(D)** Intra+P5 model in the training cohort; **(E)** Intratumoral (Intra) model in the test cohort; **(F)** Intra+P1 model in the test cohort; **(G)** Intra+P3 model in the test cohort; and **(H)** Intra+P5 model in the test cohort.

ROC analysis (**Figure 3**) showed that the Intra+P1 model performed better than the intratumoral-only model (Intra) and other peritumoral expansion models (Intra+P3 and Intra+P5). The detailed performance metrics for the Intra+P1 model in both cohorts are summarized in **Table 3**, and the results for all evaluated models are provided in **Supplementary Table 3**. The pairwise DeLong comparisons for both the ROI configurations and machine learning algorithms are summarized in **Supplementary Table 4**.

**Table 3:** Performance of different machine learning models for predicting LVSI based on the Intra+P1 radiomics features in the training and testing cohorts.

Cohort	Models	AUC (95%CI)	ACC	SEN	SPE	PPV	NPV
Training set	LR	0.866 (0.807–0.926)	0.789	0.930	0.694	0.671	0.937
	SVM	0.959 (0.930–0.988)	0.901	0.877	0.918	0.877	0.918
	RF	0.845 (0.778–0.913)	0.789	0.825	0.765	0.701	0.867
	Extra Trees	0.808 (0.734–0.883)	0.782	0.719	0.824	0.732	0.814
	LightGBM	0.914 (0.868–0.960)	0.852	0.772	0.906	0.846	0.856
	MLP	0.923 (0.879–0.967)	0.873	0.842	0.894	0.842	0.894
Testing set	LR	0.843 (0.731–0.955)	0.823	0.840	0.811	0.750	0.882
	SVM	0.722 (0.591–0.853)	0.710	0.760	0.676	0.613	0.806
	RF	0.758 (0.638–0.879)	0.726	0.680	0.757	0.654	0.778
	ExtraTrees	0.757 (0.635–0.878)	0.742	0.480	0.919	0.800	0.723
	LightGBM	0.779 (0.660–0.898)	0.774	0.720	0.811	0.720	0.811
	MLP	0.834 (0.726–0.941)	0.790	0.840	0.757	0.700	0.875

AUC, area under the receiver operating characteristic curve; ACC, accuracy; SEN, sensitivity; SPE, specificity; PPV, positive predictive value; NPV, negative predictive value; LR, logistic regression; SVM, support vector machine; RF, random forest; ExtraTrees, extremely randomized trees; LightGBM, light gradient boosting machine; MLP, multilayer perceptron.

**Figure 4:**
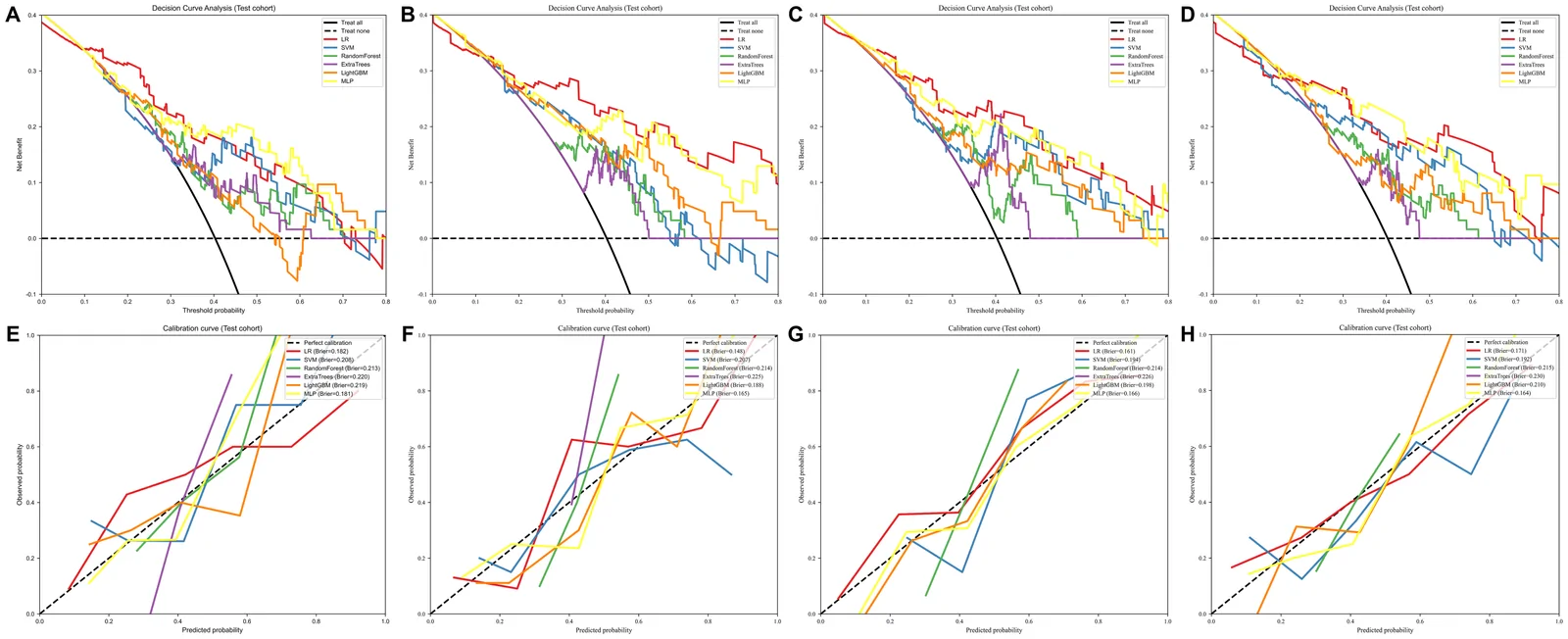
Decision curve analysis (DCA) and calibration curves of machine learning models based on different tumor regions (intratumoral and peritumoral) in the test cohort. **(A–D)** DCA curves for the Intra, Intra+P1, Intra+P3, and Intra+P5 models; **(E–H)** corresponding calibration curves for the same models.

Calibration and decision curve analyses (**Figure 4**) further indicated good agreement between the predicted probabilities and observed outcomes, as well as a favorable net clinical benefit across a wide range of threshold probabilities.

**Figure 5:**
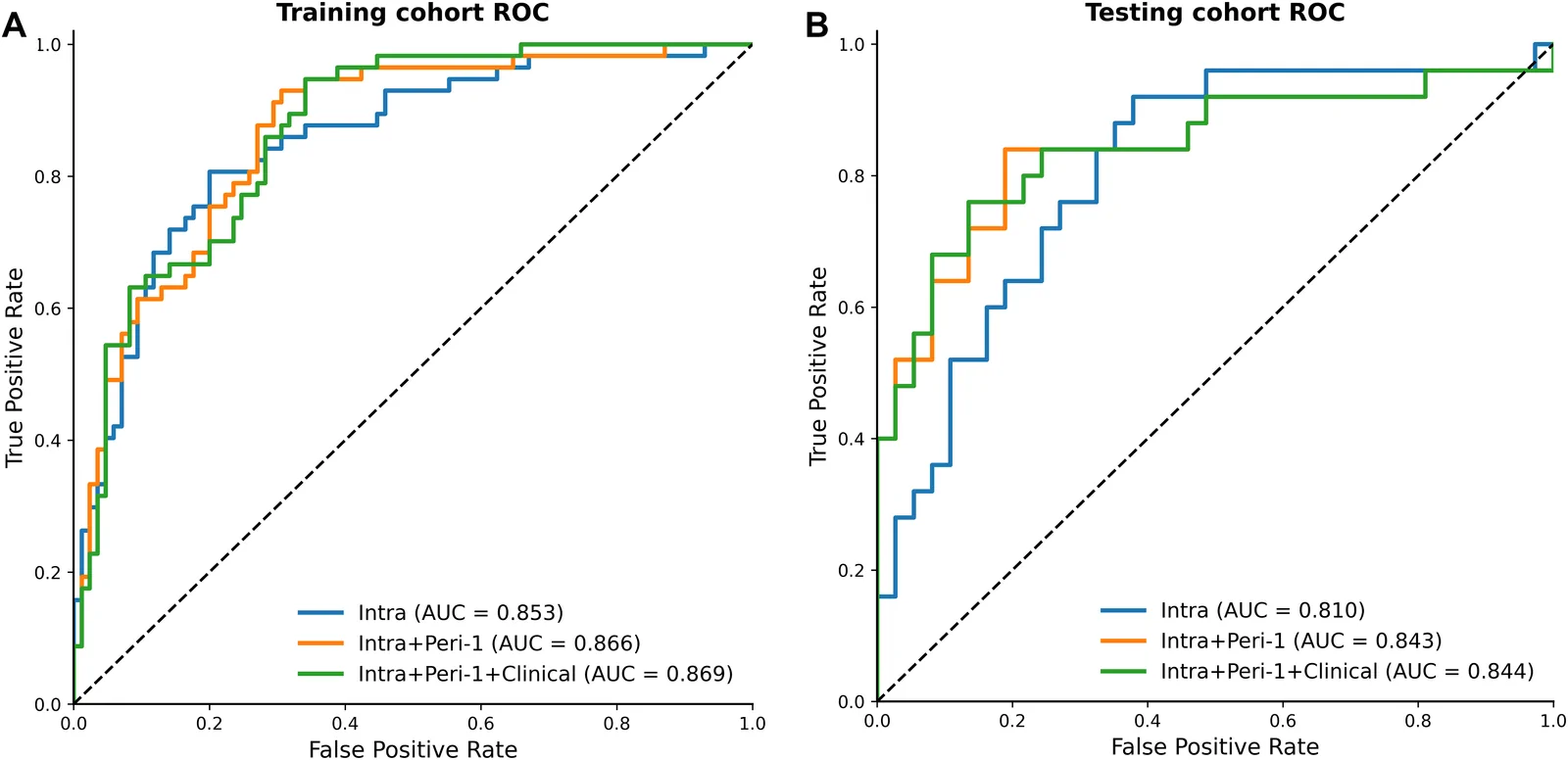
Comparison of ROC curves among the intratumoral model (Intra), the radiomics model with peritumoral expansion (Intra+P1), and the combined clinical–radiomics model (Intra+P1+Clinical) in the training and test cohorts. **(A)** Training cohort; **(B)** Testing cohort.

Based on its highest predictive performance in the current cohort, the Intra+P1 LR model was selected as the final radiomics model for subsequent interpretability analysis and model construction. A combined model integrating independent clinical predictors (maximum tumor diameter and SCC) was constructed. The combined model achieved AUCs of 0.869 and 0.844 in the training and test cohorts, respectively, without a significant improvement over the radiomics-only model (**Figure 5**).

Bootstrap sensitivity analysis was further conducted for the final Intra+P1 LR model using 1,000 resampling iterations. The mean bootstrap AUC was 0.847 (95% CI: 0.727–0.945), which was highly consistent with the original test-cohort AUC of 0.843, indicating stable model performance across repeated resampling.

### 3.4 SHAP-based interpretability and clinical application

**Figure 6:**
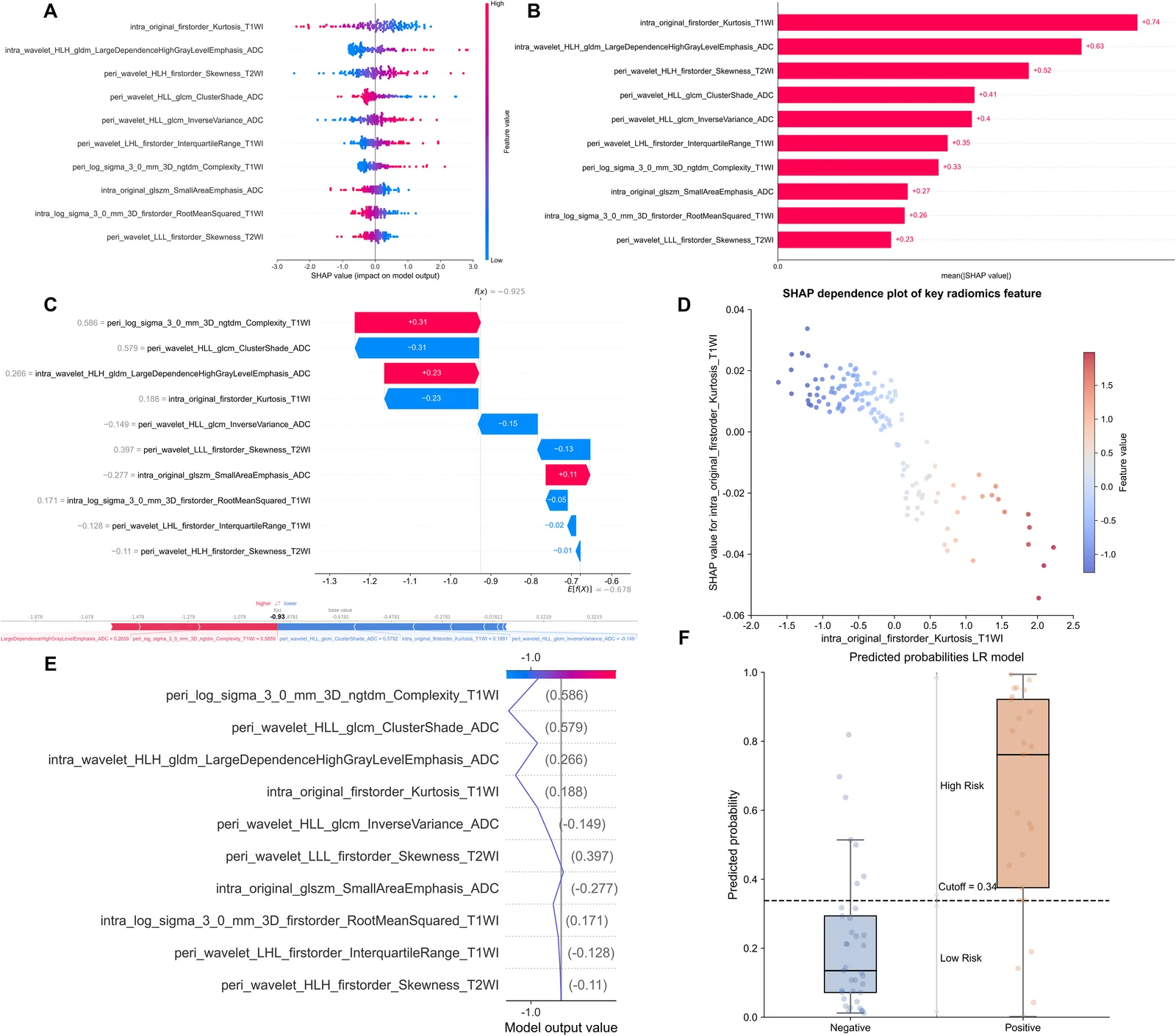
SHAP-based interpretability and clinical application analysis of the final model. **(A)** SHAP summary (beeswarm) plot showing the overall impact and direction of radiomics features on model predictions. Each point represents an individual sample, with color indicating feature value (red, high; blue, low), and the x-axis representing the SHAP value. **(B)** SHAP feature importance plot ranking features according to the mean absolute SHAP values. **(C)** SHAP waterfall plot combined with a force plot illustrating the contribution of individual features to the prediction for a representative case. **(D)** SHAP dependence plot showing the relationship between the key feature (intra_original_firstorder_Kurtosis_T1WI) and its corresponding SHAP value, with color indicating feature magnitude. **(E)** SHAP decision plot showing the cumulative contribution of features to the model output across individual samples, reflecting the decision-making process of the model. **(F)** Distribution of predicted probabilities for negative and positive cases in the test cohort. Each point represents one patient. Box-and-whisker plots summarize the distribution. The optimal cutoff value (0.34), determined using the Youden index, was used to stratify patients into low-risk and high-risk groups for LVSI, with predicted probabilities >0.34 indicating a higher likelihood of LVSI.

SHAP analysis was performed to interpret the final model at the both global and individual levels. At the global level, the SHAP summary plot (**Figure 6A**) demonstrated the relative importance and directional effects of the radiomics features on the model output. Both intratumoral and peritumoral features substantially contributed to the model predictions. The SHAP bar plot (**Figure 6B**), based on the mean absolute SHAP values, further confirmed the dominant contribution of the key features.

At the individual level, SHAP waterfall plots combined with force plots (**Figure 6C**) illustrate the cumulative contribution of each feature to the prediction for a representative case. The SHAP dependence plot (**Figure 6D**) showed an inverse association between the top-ranked feature (intra_original_firstorder_Kurtosis_T1WI) and its SHAP value, indicating that higher feature values were associated with a reduced predicted risk of LVSI. SHAP decision plots (**Figure 6E**) further demonstrated the cumulative feature effects across multiple samples, reflecting decision-making process of the model.

Finally, the distribution of predicted probabilities in the test cohort (**Figure 6F**) showed a clear separation between LVSI-positive and LVSI-negative cases, supporting the model’s ability to stratify patients into distinct risk groups. Using the optimal cutoff value determined by the Youden index (0.34), patients could be categorized into low- and high-risk groups for LVSI, potentially facilitating preoperative risk assessment and individualized treatment planning.

## 4 Discussion

In this study, we developed and internally validated an interpretable MRI-based radiomics model for the preoperative prediction of LVSI in patients with cervical cancer. Varying the extent of the peritumoral region revealed that a narrowly defined 1-mm expansion provided the most informative complement to intratumoral features. Among all configurations, the Intra+P1 model showed the best overall performance across classifiers, with logistic regression achieving stable discrimination (test AUC of 0.843). Further expansion of the peritumoral region or the addition of clinical variables did not lead to meaningful improvements in predictive performance.

A key contribution of this study is the systematic evaluation of the peritumoral extent, a methodological issue that remains insufficiently standardized in cervical cancer radiomics. The superior performance of the 1-mm peritumoral region may have a biological basis. LVSI reflects tumor cell invasion into lymphatic or vascular spaces and is therefore closely related to biological alterations at the tumor–host interface rather than being confined solely to the tumor core (4–6, 17). The immediate peritumoral microenvironment may capture invasive tumor fronts, stromal remodeling, angiogenesis, lymphangiogenesis, extracellular matrix alterations, and inflammatory interactions associated with lymphovascular dissemination (14, 18, 19). Therefore, incorporating a narrow peritumoral margin may provide complementary information beyond intratumoral heterogeneity.

In contrast, larger peritumoral expansions may progressively include greater amounts of adjacent normal cervical or uterine stromal tissue, thereby diluting tumor-related imaging signals and reducing the biological specificity of radiomics features. This may partially explain why the 1-mm peritumoral region achieved the highest predictive performance in the current cohort study. Nevertheless, the optimal peritumoral extent should be interpreted as an imaging-derived approximation rather than a strict biological boundary, and it may vary across studies because of differences in cohort composition, MRI sequences, preprocessing strategies, segmentation methods, and model development pipelines (15, 20, 21).

Previous peritumoral radiomics studies in cervical cancer have also supported the value of peritumoral information for LVSI prediction. Cui et al. evaluated multiparametric MRI-based peritumoral radiomics in early-stage cervical cancer and reported that features extracted from 3-mm and 7-mm peritumoral regions on CE-T1WI and T2WI contributed to optimal discrimination, with a validation AUC of 0.788 for the radiomics nomogram (21). Their findings support the hypothesis that peritumoral radiomics captures biologically relevant information associated with LVSI. Compared with that study, our work incorporated ADC maps in addition to T2WI and CE-T1WI, evaluated standardized 1-, 3-, and 5-mm peritumoral regions after isotropic resampling, compared multiple machine learning algorithms, and used SHAP analysis to provide model interpretability. These methodological advances enabled a more comprehensive assessment of the spatial contribution of peritumoral information to LVSI prediction and may partly explain the different optimal peritumoral extents observed in our study.

Recent studies have expanded MRI-based LVSI prediction from conventional radiomics toward habitat analysis, deep learning, and hybrid radiomics–deep learning frameworks (8, 22–25). Conventional radiomics studies have demonstrated that quantitative MRI features can provide useful information for preoperative LVSI assessment, whereas habitat-based approaches have further highlighted the importance of spatially heterogeneous tumor subregions in capturing biologically relevant information associated with tumor aggressiveness and lymphovascular dissemination (8, 23). In addition, recent multiparametric MRI-based radiomics-deep learning models have shown promising predictive performance. Yang et al. developed a comprehensive model integrating radiomics and deep learning features from T2WI, ADC, and CE-T1WI, achieving a validation AUC of 0.859, which outperformed either radiomics or deep learning alone (24). Furthermore, a recent two-center mpMRI radiomics study demonstrated robust external validation performance, reporting external validation AUCs of 0.797 for the mpMRI radiomics model and 0.847 for the combined clinical-radiomics model (25).

Compared with these previous studies, the primary focus of the present work was not solely model optimization but also the systematic investigation of peritumoral spatial extent within a standardized radiomics framework (26). By directly comparing 1-mm, 3-mm, and 5-mm peritumoral regions within a unified multiparametric MRI radiomics framework, we demonstrated that a narrow 1-mm peritumoral region provided the greatest incremental value for LVSI prediction. Moreover, SHAP analysis was incorporated to improve model interpretability and identify the most influential radiomics features associated with model predictions. This interpretable machine learning framework aims to balance predictive performance, biological plausibility, and clinical transparency, thereby facilitating clinical translation.

At the modeling level, LR demonstrated stable performance across different ROI configurations and achieved the best results in the Intra+P1 model. Although more complex machine learning algorithms can model nonlinear relationships, simpler models may offer superior generalizability and robustness, particularly in settings with limited sample sizes and stringent feature selection. In this study, the combination of ICC filtering, correlation reduction, mRMR selection, and LASSO regularization likely yielded a compact and informative feature set, thereby reducing the need for complex, nonlinear modeling.

Moreover, radiomics features inherently encode morphological and textural information that may already capture key aspects of tumor burden and aggressiveness, partially overlapping with conventional clinical indicators. In contrast, the selected clinical variables, including tumor diameter and SCC level, provided relatively coarse-grained information. This may explain why the addition of clinical variables did not lead to a meaningful improvement in predictive performance, suggesting that well-constructed radiomics signatures may, to some extent, replace the predictive value of traditional clinical factors. However, these findings should be further validated using larger, multi-institutional datasets.

The integration of SHAP further strengthened the interpretability of the model by enabling both global and case-specific explanations (27). The predominance of texture and wavelet-based features among the top contributors highlights the relevance of multiscale heterogeneity in LVSI prediction (12). Furthermore, SHAP decision plots provide a global view of the cumulative contribution of features across individual samples, offering insights into the model decision pathways. The distribution of the predicted probabilities showed a clear separation between the LVSI-positive and LVSI-negative cases, reflecting the model’s capacity to stratify patients into distinct risk groups. These results suggest the potential clinical utility of individualized risk assessment.

Notably, the SHAP dependence plot demonstrated a clear association between intra_original_firstorder_Kurtosis_T1WI and the model output. Higher feature values were generally associated with lower SHAP values, indicating a reduced contribution to the predicted probability of the LVSI. This finding suggests that intratumoral first-order intensity heterogeneity on contrast-enhanced T1-weighted imaging may contain biologically relevant information associated with LVSI status.

From a clinical perspective, the proposed model may provide a noninvasive tool for preoperative LVSI risk assessment using routinely acquired multiparametric MRI. Because LVSI is associated with lymph node metastasis, recurrence, and unfavorable prognosis (28, 29), the accurate identification of patients at increased risk for LVSI may facilitate risk stratification and individualized clinical evaluation. The probability output generated by the model could complement conventional imaging interpretation and pathological risk assessment by providing a quantitative risk estimation before surgery. Furthermore, patients classified as high risk for LVSI may warrant closer preoperative assessment and more careful postoperative surveillance. Therefore, the proposed model has potential value as a clinical decision-support tool within a multiparametric MRI–based evaluation framework.

The model provides a noninvasive and reproducible approach that can be derived from routine MRI without additional imaging requirements. Its favorable calibration and decision curve performance suggest its potential utility for risk-adapted clinical decision-making, particularly in patients with intermediate-risk disease, where management strategies remain variable (30). In addition, an interpretable framework may facilitate clinical adoption and could be integrated into decision support systems or radiology reporting workflows, pending external validation.

This study has several limitations. First, this was a single-center study comprising both retrospective and prospective cohorts, with a moderate sample size, which may limit the generalizability of the findings. Although bootstrap sensitivity analysis demonstrated stable model performance across 1,000 resampling iterations and a stratified train–test split was applied, external validation was not performed in the present study. Therefore, future multicenter studies with independent external cohorts and heterogeneous imaging platforms are warranted to further evaluate the robustness, reproducibility, and clinical applicability of the proposed model. Second, feature selection and model development were performed using the same dataset. Although multiple measures were implemented to reduce overfitting, including ICC filtering, feature reduction, regularization, and bootstrap sensitivity analysis, fully nested cross-validation or prospective validation would further strengthen the methodological rigor and reduce potential bias. Third, tumor segmentation was performed manually, which may have introduced observer variability. Although reproducibility was assessed using ICC analysis, manual delineation remains time-consuming and may affect its scalability in routine clinical practice. Future studies incorporating automated or deep learning–based segmentation may improve the segmentation consistency, workflow efficiency, and clinical applicability. Fourth, although obvious non-target structures, such as the bladder and rectal lumen, were manually excluded from the expanded peritumoral regions, anatomically contiguous adjacent tissues were retained because they may contain biologically relevant tumor–host interface information. Consequently, the biological boundaries of the peritumoral habitat cannot be fully defined using imaging techniques alone. Future studies integrating anatomy-constrained ROI generation and spatial histopathological validation may further improve biological specificity. Finally, although SHAP improves model interpretability by quantifying feature contributions, it reflects statistical associations rather than causal biological mechanisms. Therefore, the biological significance of individual radiomics features should be interpreted cautiously. Future studies should focus on multicenter validation, harmonization of imaging protocols, and integration of radiomics with complementary data sources, such as genomic biomarkers, pathological information, and deep learning–derived imaging features. Such multimodal approaches may improve predictive performance, enhance biological interpretability, and facilitate a more comprehensive characterization of tumor heterogeneity for individualized risk assessment. Longitudinal imaging analyses may provide further insights into tumor evolution and disease progression.

## 5 Conclusion

In conclusion, we developed an interpretable MRI-based radiomics model for the preoperative prediction of LVSI in cervical cancer patients. A narrowly defined 1-mm peritumoral region provided the most informative complement to intratumoral features, highlighting the role of the tumor boundary in reflecting the invasive behavior. These findings emphasize the importance of spatially informed feature extraction in radiomics and may facilitate noninvasive LVSI risk stratification and individualized clinical decision-making in patients with cervical cancer.

## Data Availability

The datasets generated and/or analyzed during the current study are not publicly available due to patient privacy and ethical restrictions. However, the datasets are available from the corresponding author on reasonable request.
