# Supplementary material for: An interpretable MRI radiomics approach for preoperative prediction of lymphovascular space invasion in cervical cancer using optimal peritumoral region
Source: Front Oncol. 2026 Aug 31;16:1859587. doi: 10.3389/fonc.2026.1859587 (PMC13573683; doi:10.3389/fonc.2026.1859587)
Supplement: Supplementary file 1 [file DataSheet1.docx]

**Supplemental Table**

**Supplementary Table 1.** Detailed MRI acquisition parameters used in this study

| **MRI** | **Philips Ingenia CX 3.0T** | **Siemens MAGNETOM Verio 3.0T** |
| --- | --- | --- |
| T1WI | TR/TE=605/10ms，thickness=4mm，FOV=240× 240mm | TR/TE=450/12ms，thickness=4mm，FOV=230×230mm |
| T2WI | TR/TE=3013/75ms，thickness=4mm，FOV=240×240mm | TR/TE=3800/96ms，thickness=5mm，FOV=230×230mm |
| T1WI pre-contrast | TR/TE=5.5/2ms，thickness=3mm，FOV=240×240mm | TR/TE=3.91/1.89ms，thickness=3mm，FOV=380×261mm |
| T1WI dynamic contrast | TR/TE=5.5/2ms，thickness=3mm，FOV=240×240mm | TR/TE=3.91/1.89ms，thickness=3mm，FOV=380×261mm |
| DWI | TR/TE=6000/49ms，thickness=4mm，FOV=200×300mm | TR/TE=4387/68.8ms，thickness=4mm，FOV=306×239mm |

**Supplementary Table 2. Top 20 radiomics features for each model**

(A) **Intra**

| **Rank** | **Feature** | **Region** | **Sequence** |
| --- | --- | --- | --- |
| 1 | log_sigma_3_0_mm_3D_firstorder_Skewness | intra | T1WI |
| 2 | original_glszm_SmallAreaEmphasis | intra | ADC |
| 3 | original_firstorder_Kurtosis | intra | T1WI |
| 4 | wavelet_HLH_gldm_LargeDependenceHighGrayLevelEmphasis | intra | ADC |
| 5 | wavelet_LHH_glszm_LargeAreaHighGrayLevelEmphasis | intra | T1WI |
| 6 | log_sigma_3_0_mm_3D_firstorder_Minimum | intra | T1WI |
| 7 | wavelet_LHL_firstorder_Range | intra | T1WI |
| 8 | log_sigma_5_0_mm_3D_firstorder_Minimum | intra | T1WI |
| 9 | wavelet_HLL_glcm_ClusterProminence | intra | T1WI |
| 10 | wavelet_HLL_firstorder_Maximum | intra | T1WI |
| 11 | wavelet_HLH_glszm_SmallAreaEmphasis | intra | ADC |
| 12 | wavelet_HHL_glcm_ClusterShade | intra | T2WI |
| 13 | wavelet_HLH_firstorder_Kurtosis | intra | ADC |
| 14 | wavelet_HHL_firstorder_RootMeanSquared | intra | ADC |
| 15 | wavelet_HHL_glszm_GrayLevelVariance | intra | T2WI |
| 16 | wavelet_LHL_glcm_ClusterProminence | intra | ADC |
| 17 | wavelet_LLH_glszm_ZoneVariance | intra | T2WI |
| 18 | wavelet_HHH_firstorder_90Percentile | intra | T2WI |
| 19 | wavelet_HLL_glcm_ClusterProminence | intra | ADC |
| 20 | wavelet_HLH_ngtdm_Busyness | intra | T2WI |

(B) **Intra+P1**

| **Rank** | **Feature** | **Region** | **Sequence** |
| --- | --- | --- | --- |
| 1 | log_sigma_3_0_mm_3D_firstorder_Skewness | intra | T1WI |
| 2 | wavelet_LHL_firstorder_InterquartileRange | peri | T1WI |
| 3 | wavelet_HLL_glcm_ClusterShade | peri | ADC |
| 4 | log_sigma_3_0_mm_3D_ngtdm_Complexity | peri | T1WI |
| 5 | wavelet_HLL_glcm_InverseVariance | peri | ADC |
| 6 | wavelet_HLH_gldm_LargeDependenceHighGrayLevelEmphasis | intra | ADC |
| 7 | log_sigma_3_0_mm_3D_glszm_SmallAreaHighGrayLevelEmphasis | peri | T1WI |
| 8 | wavelet_HLH_firstorder_Kurtosis | intra | ADC |
| 9 | original_glszm_GrayLevelVariance | peri | T1WI |
| 10 | log_sigma_3_0_mm_3D_firstorder_RootMeanSquared | intra | T1WI |
| 11 | log_sigma_3_0_mm_3D_firstorder_Minimum | peri | T1WI |
| 12 | wavelet_HHH_firstorder_Kurtosis | peri | ADC |
| 13 | original_firstorder_Kurtosis | intra | T1WI |
| 14 | wavelet_LHH_firstorder_Kurtosis | peri | ADC |
| 15 | wavelet_HHL_firstorder_RootMeanSquared | peri | T2WI |
| 16 | wavelet_LHH_glszm_LargeAreaHighGrayLevelEmphasis | intra | T1WI |
| 17 | original_glszm_SmallAreaEmphasis | intra | ADC |
| 18 | wavelet_LLL_firstorder_Skewness | peri | T2WI |
| 19 | wavelet_HLL_glcm_ClusterProminence | intra | ADC |
| 20 | wavelet_HLH_firstorder_Skewness | peri | T2WI |

(C) **Intra+P3**

| **Rank** | **Feature** | **Region** | **Sequence** |
| --- | --- | --- | --- |
| 1 | log_sigma_3_0_mm_3D_firstorder_Skewness | intra | T1WI |
| 2 | wavelet_LHH_firstorder_Skewness | peri | ADC |
| 3 | wavelet_HLH_gldm_LargeDependenceHighGrayLevelEmphasis | intra | ADC |
| 4 | original_glszm_SmallAreaEmphasis | intra | ADC |
| 5 | log_sigma_3_0_mm_3D_glszm_SmallAreaHighGrayLevelEmphasis | peri | T1WI |
| 6 | wavelet_LLH_firstorder_Kurtosis | peri | T1WI |
| 7 | original_firstorder_Kurtosis | intra | T1WI |
| 8 | log_sigma_3_0_mm_3D_glcm_ClusterShade | peri | T2WI |
| 9 | original_shape_Flatness | peri | ADC |
| 10 | wavelet_LLH_firstorder_Skewness | peri | T1WI |
| 11 | log_sigma_5_0_mm_3D_firstorder_Minimum | intra | T1WI |
| 12 | log_sigma_5_0_mm_3D_ngtdm_Complexity | peri | T1WI |
| 13 | wavelet_HHL_glcm_ClusterShade | intra | T2WI |
| 14 | wavelet_LHL_firstorder_Maximum | peri | T1WI |
| 15 | wavelet_LHH_glszm_LargeAreaHighGrayLevelEmphasis | intra | T1WI |
| 16 | original_firstorder_Skewness | peri | T2WI |
| 17 | log_sigma_5_0_mm_3D_glcm_DifferenceEntropy | peri | ADC |
| 18 | wavelet_HHL_firstorder_RootMeanSquared | intra | ADC |
| 19 | wavelet_LLL_glcm_DifferenceVariance | peri | T1WI |
| 20 | wavelet_LHL_glcm_ClusterProminence | peri | T1WI |

(D) **Intra+P5**

| **Rank** | **Feature** | **Region** | **Sequence** |
| --- | --- | --- | --- |
| 1 | log_sigma_3_0_mm_3D_firstorder_Skewness | intra | T1WI |
| 2 | wavelet_HLH_gldm_LargeDependenceHighGrayLevelEmphasis | intra | ADC |
| 3 | wavelet_HHL_glcm_ClusterShade | peri | T2WI |
| 4 | wavelet_LLH_firstorder_Kurtosis | peri | T1WI |
| 5 | wavelet_LHL_firstorder_90Percentile | peri | T1WI |
| 6 | original_firstorder_Kurtosis | intra | T1WI |
| 7 | wavelet_LHH_firstorder_Kurtosis | peri | ADC |
| 8 | log_sigma_5_0_mm_3D_glcm_DifferenceVariance | peri | T1WI |
| 9 | wavelet_LLL_firstorder_Minimum | peri | T1WI |
| 10 | original_glszm_SmallAreaEmphasis | intra | ADC |
| 11 | log_sigma_5_0_mm_3D_firstorder_Minimum | intra | T1WI |
| 12 | log_sigma_3_0_mm_3D_firstorder_Minimum | intra | T1WI |
| 13 | wavelet_LHH_firstorder_Kurtosis | peri | T2WI |
| 14 | wavelet_HLL_firstorder_Skewness | peri | ADC |
| 15 | wavelet_HHL_glcm_ClusterShade | intra | T2WI |
| 16 | wavelet_LLH_gldm_LargeDependenceHighGrayLevelEmphasis | peri | ADC |
| 17 | wavelet_HHL_firstorder_RootMeanSquared | intra | ADC |
| 18 | wavelet_HLH_glszm_SmallAreaLowGrayLevelEmphasis | peri | T1WI |
| 19 | original_shape_Flatness | peri | ADC |
| 20 | log_sigma_3_0_mm_3D_glcm_ClusterShade | peri | T2WI |

**Supplementary Table 3.** Performance of different machine learning models across ROI configurations in the training and test cohorts

(A) **Intra**

| **Model** | **Task** | **Accuracy** | **AUC** | **95% CI** | **Sensitivity** | **Specificity** | **PPV** | **NPV** | **F1** | **Threshold** |
| --- | --- | --- | --- | --- | --- | --- | --- | --- | --- | --- |
| LR | Train | 0.803 | 0.853 | 0.789 - 0.918 | 0.807 | 0.800 | 0.730 | 0.861 | 0.767 | 0.409 |
| LR | Test | 0.742 | 0.810 | 0.698 - 0.922 | 0.920 | 0.622 | 0.622 | 0.920 | 0.742 | 0.230 |
| SVM | Train | 0.866 | 0.933 | 0.893 - 0.973 | 0.825 | 0.894 | 0.839 | 0.884 | 0.832 | 0.394 |
| SVM | Test | 0.774 | 0.716 | 0.573 - 0.859 | 0.600 | 0.892 | 0.789 | 0.767 | 0.682 | 0.489 |
| RF | Train | 0.838 | 0.891 | 0.837 - 0.946 | 0.789 | 0.871 | 0.804 | 0.860 | 0.796 | 0.415 |
| RF | Test | 0.694 | 0.701 | 0.567 - 0.834 | 0.520 | 0.811 | 0.650 | 0.714 | 0.578 | 0.478 |
| ExtraTrees | Train | 0.789 | 0.835 | 0.765 - 0.904 | 0.737 | 0.824 | 0.737 | 0.824 | 0.737 | 0.400 |
| ExtraTrees | Test | 0.710 | 0.710 | 0.580 - 0.841 | 0.400 | 0.919 | 0.769 | 0.694 | 0.526 | 0.477 |
| LightGBM | Train | 0.782 | 0.856 | 0.795 - 0.917 | 0.754 | 0.800 | 0.717 | 0.829 | 0.735 | 0.401 |
| LightGBM | Test | 0.629 | 0.665 | 0.526 - 0.804 | 0.680 | 0.595 | 0.531 | 0.733 | 0.596 | 0.408 |
| MLP | Train | 0.817 | 0.877 | 0.819 - 0.935 | 0.789 | 0.835 | 0.763 | 0.855 | 0.776 | 0.423 |
| MLP | Test | 0.790 | 0.797 | 0.676 - 0.918 | 0.640 | 0.892 | 0.800 | 0.786 | 0.711 | 0.492 |

(B) **Intra+P1**

| **Model** | **Task** | **Accuracy** | **AUC** | **95% CI** | **Sensitivity** | **Specificity** | **PPV** | **NPV** | **F1** | **Threshold** |
| --- | --- | --- | --- | --- | --- | --- | --- | --- | --- | --- |
| LR | Train | 0.789 | 0.866 | 0.807 - 0.926 | 0.930 | 0.694 | 0.671 | 0.937 | 0.779 | 0.278 |
| LR | Test | 0.823 | 0.843 | 0.731 - 0.955 | 0.840 | 0.811 | 0.750 | 0.882 | 0.792 | 0.338 |
| SVM | Train | 0.901 | 0.959 | 0.930 - 0.988 | 0.877 | 0.918 | 0.877 | 0.918 | 0.877 | 0.413 |
| SVM | Test | 0.710 | 0.722 | 0.591 - 0.853 | 0.760 | 0.676 | 0.613 | 0.806 | 0.679 | 0.469 |
| RF | Train | 0.789 | 0.845 | 0.778 - 0.913 | 0.825 | 0.765 | 0.701 | 0.867 | 0.758 | 0.400 |
| RF | Test | 0.726 | 0.758 | 0.638 - 0.879 | 0.680 | 0.757 | 0.654 | 0.778 | 0.667 | 0.431 |
| ExtraTrees | Train | 0.782 | 0.808 | 0.734 - 0.883 | 0.719 | 0.824 | 0.732 | 0.814 | 0.726 | 0.403 |
| ExtraTrees | Test | 0.742 | 0.757 | 0.635 - 0.878 | 0.480 | 0.919 | 0.800 | 0.723 | 0.600 | 0.427 |
| LightGBM | Train | 0.852 | 0.914 | 0.868 - 0.960 | 0.772 | 0.906 | 0.846 | 0.856 | 0.807 | 0.480 |
| LightGBM | Test | 0.774 | 0.779 | 0.660 - 0.898 | 0.720 | 0.811 | 0.720 | 0.811 | 0.720 | 0.500 |
| MLP | Train | 0.873 | 0.923 | 0.879 - 0.967 | 0.842 | 0.894 | 0.842 | 0.894 | 0.842 | 0.414 |
| MLP | Test | 0.790 | 0.834 | 0.726 - 0.941 | 0.840 | 0.757 | 0.700 | 0.875 | 0.764 | 0.441 |

(C) **Intra+P3**

| **Model** | **Task** | **Accuracy** | **AUC** | **95% CI** | **Sensitivity** | **Specificity** | **PPV** | **NPV** | **F1** | **Threshold** |
| --- | --- | --- | --- | --- | --- | --- | --- | --- | --- | --- |
| LR | Train | 0.824 | 0.860 | 0.795 - 0.924 | 0.649 | 0.941 | 0.881 | 0.800 | 0.747 | 0.580 |
| LR | Test | 0.806 | 0.836 | 0.729 - 0.942 | 0.760 | 0.838 | 0.760 | 0.838 | 0.760 | 0.391 |
| SVM | Train | 0.908 | 0.933 | 0.883 - 0.983 | 0.877 | 0.929 | 0.893 | 0.919 | 0.885 | 0.378 |
| SVM | Test | 0.790 | 0.749 | 0.611 - 0.888 | 0.640 | 0.892 | 0.800 | 0.786 | 0.711 | 0.506 |
| RF | Train | 0.775 | 0.831 | 0.761 - 0.902 | 0.789 | 0.765 | 0.692 | 0.844 | 0.738 | 0.393 |
| RF | Test | 0.758 | 0.716 | 0.583 - 0.849 | 0.480 | 0.946 | 0.857 | 0.729 | 0.615 | 0.484 |
| ExtraTrees | Train | 0.789 | 0.828 | 0.759 - 0.898 | 0.772 | 0.800 | 0.721 | 0.840 | 0.746 | 0.403 |
| ExtraTrees | Test | 0.790 | 0.798 | 0.684 - 0.912 | 0.720 | 0.838 | 0.750 | 0.816 | 0.735 | 0.413 |
| LightGBM | Train | 0.831 | 0.877 | 0.817 - 0.936 | 0.719 | 0.906 | 0.837 | 0.828 | 0.774 | 0.480 |
| LightGBM | Test | 0.742 | 0.744 | 0.615 - 0.873 | 0.560 | 0.865 | 0.737 | 0.744 | 0.636 | 0.496 |
| MLP | Train | 0.831 | 0.867 | 0.802 - 0.932 | 0.789 | 0.859 | 0.789 | 0.859 | 0.789 | 0.448 |
| MLP | Test | 0.790 | 0.822 | 0.716 - 0.928 | 0.720 | 0.838 | 0.750 | 0.816 | 0.735 | 0.489 |

(D) **Intra+P5**

| **Model** | **Task** | **Accuracy** | **AUC** | **95% CI** | **Sensitivity** | **Specificity** | **PPV** | **NPV** | **F1** | **Threshold** |
| --- | --- | --- | --- | --- | --- | --- | --- | --- | --- | --- |
| LR | Train | 0.852 | 0.882 | 0.823 - 0.940 | 0.754 | 0.918 | 0.860 | 0.848 | 0.804 | 0.543 |
| LR | Test | 0.774 | 0.800 | 0.675 - 0.925 | 0.840 | 0.730 | 0.677 | 0.871 | 0.750 | 0.308 |
| SVM | Train | 0.908 | 0.948 | 0.910 - 0.986 | 0.912 | 0.906 | 0.867 | 0.939 | 0.889 | 0.392 |
| SVM | Test | 0.774 | 0.758 | 0.626 - 0.890 | 0.640 | 0.865 | 0.762 | 0.780 | 0.696 | 0.534 |
| RF | Train | 0.746 | 0.799 | 0.722 - 0.875 | 0.754 | 0.741 | 0.662 | 0.818 | 0.705 | 0.409 |
| RF | Test | 0.677 | 0.723 | 0.597 - 0.850 | 0.800 | 0.595 | 0.571 | 0.815 | 0.667 | 0.405 |
| ExtraTrees | Train | 0.739 | 0.793 | 0.715 - 0.872 | 0.719 | 0.753 | 0.661 | 0.800 | 0.689 | 0.407 |
| ExtraTrees | Test | 0.710 | 0.724 | 0.599 - 0.850 | 0.520 | 0.838 | 0.684 | 0.721 | 0.591 | 0.425 |
| LightGBM | Train | 0.810 | 0.881 | 0.823 - 0.939 | 0.842 | 0.788 | 0.727 | 0.882 | 0.780 | 0.411 |
| LightGBM | Test | 0.726 | 0.713 | 0.579 - 0.847 | 0.560 | 0.838 | 0.700 | 0.738 | 0.622 | 0.498 |
| MLP | Train | 0.859 | 0.912 | 0.865 - 0.959 | 0.737 | 0.941 | 0.894 | 0.842 | 0.808 | 0.550 |
| MLP | Test | 0.774 | 0.813 | 0.699 - 0.927 | 0.800 | 0.757 | 0.690 | 0.848 | 0.741 | 0.441 |

**Supplementary Table 4.** Pairwise DeLong test comparisons of ROC AUCs between the Intra+P1 LR model and competing ROI configurations and machine learning algorithms

| **Comparison** | **Reference AUC** | **Comparator AUC** | **AUC difference** | **P value** |
| --- | --- | --- | --- | --- |
| **ROI comparisons** |  |  |  |  |
| Intra+P1 LR vs Intra LR | 0.843 | 0.810 | 0.034 | 0.450 |
| Intra+P1 LR vs Intra+P3 LR | 0.843 | 0.836 | 0.008 | 0.847 |
| Intra+P1 LR vs Intra+P5 LR | 0.843 | 0.800 | 0.043 | 0.501 |
| **Algorithm comparisons (Intra+P1)** |  |  |  |  |
| LR vs SVM | 0.843 | 0.722 | 0.121 | 0.004 |
| LR vs Random Forest | 0.843 | 0.758 | 0.085 | 0.109 |
| LR vs Extra Trees | 0.843 | 0.757 | 0.086 | 0.129 |
| LR vs LightGBM | 0.843 | 0.779 | 0.064 | 0.205 |
| LR vs MLP | 0.843 | 0.834 | 0.010 | 0.732 |
